# Biocompatibility of Cyclopropylamine-Based Plasma Polymers Deposited at Sub-Atmospheric Pressure on Poly (ε-caprolactone) Nanofiber Meshes

**DOI:** 10.3390/nano9091215

**Published:** 2019-08-28

**Authors:** Ke Vin Chan, Mahtab Asadian, Iuliia Onyshchenko, Heidi Declercq, Rino Morent, Nathalie De Geyter

**Affiliations:** 1 Research Unit Plasma Technology (RUPT), Department of Applied Physics, Faculty of Engineering and Architecture, Ghent University, Sint-Pieternieuwstraat 41, 9000 Ghent, Belgium; 2 Tissue Engineering Group, Department of Human Structure and Repair, Faculty of Medicine and Health Sciences, Ghent University, De Pintelaan 185, B3, B-9000 Ghent, Belgium

**Keywords:** biomaterials, non-thermal plasma, plasma polymerisation, nanofibers, cell adhesion

## Abstract

In this work, cyclopropylamine (CPA) monomer was plasma-polymerized on poly (ε-caprolactone) nanofiber meshes using various deposition durations to obtain amine-rich surfaces in an effort to improve the cellular response of the meshes. Scanning electron microscopy and X-ray photoelectron spectroscopy (XPS) were used to investigate the surface morphology and surface chemical composition of the PCL samples, respectively. The measured coating thickness was found to linearly increase with deposition duration at a deposition rate of 0.465 nm/s. XPS analysis revealed that plasma exposure time had a considerable effect on the surface N/C and O/C ratio as well as on amino grafting efficiency and amino selectivity. In addition, cell studies showed that cell adhesion and proliferation significantly improved for all coated samples.

## 1. Introduction

Scaffolds consisting of nanofibers (NFs) (fiber diameter < 500 nm) have been extensively used for tissue engineering research because they closely mimic the structural properties of the extracellular matrix (ECM) [1,2,3,4,5]. NFs for tissue engineering purposes are typically made from either synthetic polymers such as poly (ε-caprolactone) (PCL) [6,7], poly(lactic acid) (PLA) [8], poly(urethane) (PU) [9], polyethersulfone (PES) [10] and others or naturally occurring polymers such as gelatine [11], silk fibroin [12], chitosan [13] and others. However, it is also possible to use a mixture of both polymer types (or to use specific additives) typically leading to the manufacturing of hybrid NF scaffolds with advantages of each polymer component [14,15]. Although there are many manufacturing methodologies to produce NF scaffolds from polymers for tissue engineering purposes, electrospinning is by far the most widely utilized due to its simplicity and consistency.

Amongst the wide range of polymers mentioned above that can be used for electrospinning, PCL is extensively applied since it is well known to be biodegradable and non-cytotoxic, which are desirable properties for tissue engineering scaffolds [14,16,17]. However, this polymer also belongs to the material group with hydrophobic surface characteristics and thus performs poorly in terms of cell adhesion due to the lack of surface functionality [18]. This disadvantage has prompted many investigations on various methods to improve the wettability properties of PCL NF scaffolds by surface modification and functionalization such as wet chemical treatment, non-thermal plasma treatment, UV exposure, etc. [7,19,20]. One of the most suitable and widely used techniques to achieve this goal is via plasma activation, which is known to introduce polar functional surface groups such as hydroxyls, carboxylic acids, amino groups, etc. [6,7,21,22,23,24]. Furthermore, this technique does not alter the morphology and bulk properties of the NF samples and typically greatly enhances surface biocompatibility. For instance, it has been reported in literature that an air plasma activation step has shown to significantly improve cell adhesion and proliferation of primary porcine smooth muscle cells, Schwann cells and human foetal osteoblast cells on PCL NF scaffolds resulting from the incorporation of surface oxygen functional groups [6,14,25]. Moreover, two consistent observations were also revealed within these studies: (1) the cells were found to be more spread out and elongated on the plasma-treated surfaces and (2) after prolonged cell culture duration, a cell monolayer could be seen on the plasma-activated PCL NF scaffolds. Another study applying plasma activation on PCL NFs using argon and oxygen as discharge gases demonstrated that the plasma-modified PCL surfaces have significantly improved biocompatibility across multiple model cell lines (fibroblasts, chondrocytes and osteoblasts) due to the increased surface hydrophilicity resulting from the incorporated polar functional groups [26,27,28]. Thus, the surface functionality of PCL NFs is highly important as it has a critical role in promoting cellular interactions.

Additionally, plasma-activated PCL NF scaffolds can also be subsequently used for specific protein grafting such as laminin, collagen and gelatin to further improve biocompatibility or to elicit specific cellular responses [21,22,24]. For example, Jia et al. [23] grafted soluble eggshell membrane on PCL NFs to enhance interactions between the NF surfaces and fibroblasts. In this particular study, the cells were found to infiltrate deeper into the protein-grafted scaffolds and certain gene expression was also promoted on these scaffolds. Other scaffolds such as PLA NFs have also been grafted with collagen after performing a plasma activation step, which was found to increase alkaline phosphatase activity (ALP) and mineralization of NF scaffolds by calcium deposition, which are markers for the differentiation of stem cells into osteoblasts [29]. It can thus be concluded that plasma-activated NF scaffolds can be easily adapted to the specific requirements of tissue engineering by efficient grafting of desired proteins.

Although plasma activation is a simple and effective method for NF surface modification, an important drawback, the aging affect (or so-called hydrophobic recovery), needs to be considered. This aging behaviour is caused by the high chain mobility of the polymeric molecules causing a gradual rotation of the incorporated hydrophilic polar groups inwards to the material bulk while exposing hydrophobic non-polar groups to the environment [30]. To overcome this phenomenon, plasma polymerization can be applied, resulting in the deposition of a thin polymer film, which is typically achieved by sustaining a He or Ar plasma with the addition of a precursor containing the desired surface functional groups. For example, Feng et al. [7] plasma polymerized acrylic acid on PCL NFs to attain a thin plasma polymer film (PPF) rich in carboxylic groups, which in turn resulted in a significantly improved adhesion and proliferation of pre-osteoblast cells on the PCL NFs. Although no collagen was grafted in this particular study, an increase in ALP activity was also observed. Instead of fabricating a carboxylic-rich PPF, Manakhov et al. [31] deposited an amine-rich thin PPF using cyclopropylamine (CPA) as a precursor, resulting in a substantially increased proliferation rate of C2C12 cells. Moreover, it was shown that the cells strongly adhered to plasma-polymerized PCL NF substrates as the cells did not even detach with increased trypsin concentration and time of trypsination [32]. 

Although improving the surface hydrophilicity by incorporating polar functional surface groups does improve surface biocompatibility, the degree of improvement significantly varies depending on the type of incorporated functional group. This phenomenon was observed by Lee et al. [27] who compared the effects different surfaces functionalized with a single type of functional group had on cell-surface interactions. These researchers found that amine-functionalized surfaces were superior in improving cell adhesion compared to other types of functional groups. This peculiar behaviour was explained by the fact that, under the physiological pH of cell culture, the amine groups are protonated and thus positively charged. Since glycoproteins such as fibronectin and vitronectin present in protein serum are negatively charged, these proteins are thus electrostatically immobilized on the amine-rich surfaces when introducing these in the cell culture medium. This in turn promotes cell adhesion by binding with integrin on the cell membranes [33,34]. Besides being more effective in immobilizing glycoproteins, the positively charged amine groups have also shown to upregulate osteogenesis-related genes/proteins expression while downregulating inducible nitric oxide synthase, which inhibits osteogenic differentiation in mesenchymal stem cells [35]. 

Taking into account the above-mentioned discussion, it is important to emphasize that the majority of reported plasma treatments on NFs in literature are applied for surface activation and those plasma treatments are typically performed at low pressure (< 100 Pa) [36,37]. Consequently, there are only a few articles focusing on the deposition of PPFs on NFs as in this particular case the preservation of the nanomorphology of the fibrous scaffolds, which is crucial for tissue engineering applications, is more difficult. To contribute to this rather unexplored research field, this work focusses on the deposition of an amine-rich PPF on PCL NFs to improve cell-surface interactions. The plasma polymerization process is conducted at sub-atmospheric pressure (50 kPa) in an effort to raise the plasma polymerisation process pressure closer to atmospheric conditions. Plasma polymerization is achieved using a parallel plate dielectric barrier discharge (DBD) sustained in a mixture of argon and CPA. The obtained PPFs are profoundly characterized by scanning electron microscopy (SEM) and X-ray photoelectron spectroscopy (XPS) to study changes in NF surface morphology and surface chemical functionality, respectively. Furthermore, cell adhesion and proliferation on the plasma-polymerized PCL NFs are investigated using human foreskin fibroblasts (HHFs) as model cell line.

## 2. Materials and Methods

### 2.1. Fabrication of NFs and Plasma Polymer Deposition

Prior to electrospinning, a PCL polymer solution was prepared according to a previously optimized procedure: (1) 1 part acetic acid (Sigma-Aldrich, Steinhem, Germany) was mixed with nine parts formic acid (Sigma-Aldrich, Steinhem, Germany); (2) 14% (w/v) PCL granules (average molecular weight: 40000–60000 g/mol; Sigma-Aldrich, Steinhem, Germany) were then added to the solvent mixture and (3) the solution was stirred until complete dissolution of the PCL granules [5]. In a next step, the prepared PCL solution was electrospun onto glass coverslips with a diameter of 12 mm according to the protocol described in detail in previous work [5]. Briefly, the polymer solution was electrospun using a bottom-up electrospinning process making use of a Nanospinner 24 (Inovenso, Istanbul, Turkey) device. The conducted electrospinning process resulted in the fabrication of nanofibrous meshes consisting of smooth, randomly oriented PCL NFs. 

After electrospinning, the fabricated PCL NFs were subjected to a plasma polymerization procedure, which has already been described in detail in a previous study [38]. Briefly, a stainless steel plasma chamber, containing a high-voltage top mesh electrode and a circular plate grounded bottom electrode covered with an Al_2_O_3_ layer as dielectric barrier, was pumped down to a base pressure of 0.26 Torr (35 Pa) using a rotary vane pump (Edwards- RV3, Clevedon, UK) after placing the PCL NFs on the grounded electrode. The gas gap between both electrodes was maintained at 1 mm during all experiments. Argon gas (Air Liquide, Alphagaz 1, Liege, Belgium) was then introduced into the plasma chamber through the top mesh electrode at a gas flow rate of 4 standard litre per minute (slm) making use of a Bronkhorst El-Flow^®^ mass flow controller until a chamber pressure of 375 Torr (50 kPa) was reached. This medium discharge pressure was chosen as it is the highest attainable discharge pressure at which discharge homogeneity and detrimental plasma arcing does not occur. In a next step, the chamber pressure of 50 kPa was maintained by using a fixed argon flow rate of 0.4 slm and an adjusted needle valve placed in front of the rotary vane pump. After stabilizing the chamber pressure, CPA (Acros Organics, Geel, Belgium) vapour was added to the Ar flow at a fixed flow rate of 0.125 g/h using a combination of a Bronkhorst controlled evaporation and mixing module (CEM^®^, type W-202A) and a Bronkhorst mini CORI-FLOW^®^ meter (type M12P) from Ruurlo, Netherlands. This will yield a monomer concentration of 0.005 g/l in the plasma chamber. Subsequently, the AC high voltage power source (fixed frequency of 50 kHz) was switched on and plasma polymerization was performed at a fixed discharge power of 20 W for time durations varying between 10 s and 360 s. The plasma-polymerized PCL NFs will be abbreviated in this paper as PPF-NFs.

### 2.2. XPS Analysis

XPS surface analysis of the NFs samples has been performed on a PHI Versaprobe II spectrometer (Chigasaki, Japan) employing a monochromatic Al K_α_ X-ray source (hν = 1486.6 eV) operating at 43.5 W. In an effort to minimize the effects of post-plasma oxidation processes, the time lag between the deposition of the PPFs and the introduction of the samples into the XPS intro chamber was maintained below 1 hour. All measurements were conducted in a vacuum of at least 10^−6^ Pa and the photoelectrons were detected with a hemispherical analyser positioned at an angle of 45° with respect to the normal of the sample surface. Survey scans and individual high resolution spectra (O1s, C1s, and N1s) were recorded with a pass energy of 187.85 eV and 23.5 eV with an eV step of 0.8 and 0.1, respectively. Elements present on the PCL surfaces were identified from XPS survey scans, which have been performed on five different point locations per sample condition. The obtained elements were quantified with Multipak software using a Shirley background and applying the relative sensitivity factors supplied by the manufacturer of the instrument. Multipak software was also used to curve fit the high resolution C1s peaks. First, the hydrocarbon component of the C1s spectrum (285.0 eV) was used to calibrate the energy scale. In a subsequent step, the peaks were deconvoluted using Gaussian–Lorentzian peak shapes and the full-width at half maximum (FWHM) of each line shape was constrained below 1.8 eV.

In case of CPA plasma polymers, where several nitrogen- and oxygen (due to surface oxidation)-containing groups are present [38], a precise functional group identification and quantification is very hard due to overlapping contributions in the XPS spectra. For example, primary, secondary and tertiary amines are not readily distinguishable in high resolution C1s spectra, while a strong overlap also occurs between C–N, C=N and C≡N groups in N1s spectra [39,40]. To overcome this difficulty, an advanced surface analysis technique combining XPS and chemical derivatization was also used in this work to enable a meaningful quantification of the primary amine functional groups. In this study, PPF-NFs were chemically derivatized by exposing the as-deposited plasma coatings to 4-trifluoromethyl-benzaldehyde (TFBA) (Sigma-Aldrich, Steinhem, Germany) vapour in a small glass chamber (pumped to a residual pressure of 7.5 Torr (1 kPa)) at room temperature for 40 minutes. TFBA was chosen since it is known to selectively react with NH_2_ groups, resulting in the incorporation of CF_3_ groups on the plasma deposits [38,41,42]. After the derivatization step, XPS survey scans were measured on the PCL samples using the same experimental parameters as previously mentioned. After quantifying the obtained elements with Multipak software, the amino selectivity and amino grafting efficiency of the deposits were determined using the following formulae [38,41,42]:

(1) Amino selectivity %= NH2N=F3N × 100,

(2) Amino grafting efficiency %= NH2C=F3C−8F3 × 100,

### 2.3. SEM Imaging

The morphology of pristine PCL NFs and PPF-NFs was visualized using a JSM-6010 PLUS/LV SEM device after gold sputtering the samples with a JFC-1300 Auto Coater (JEOL, Tokyo, Japan) for 25 s. To obtain the SEM images shown in this work, an accelerating voltage varying between 5 kV and 20 kV, a filament temperature of 160 °C and an electron beam intensity of approximately 95 µA was used. The average diameter of 25 PCL NFs with (*D_w_*) and without (*D_u_*), and a deposited PPF was also measured with ImageJ software. Using these PCL NF diameter measurements, the thickness of the deposited coatings using deposition times between 10 and 360 s was calculated using the following equation:

(3) $$Film\;thickness=\;\frac{Average\left(D_{w}\right)-Average\left(D_{u}\right)}{2}$$

### 2.4. Cell Culturing

Prior to cell culturing, the NFs and PPF-NFs samples were first sterilized by exposure to UV light for 30 min. Afterwards, HFF cells were seeded onto the samples in a 24-well plate at a density of 40,000 cells/mL. Subsequently, 1 mL of the cell suspension media was added to each well plate. Cell culturing was performed using Dulbecco’s modified Eagle’s medium (DMEM) with glutamax (Gibco Invitrogen, Grand Island, NY, USA) supplemented with 15% fetal calf serum (Gibco Invitrogen, Grand Island, NY, USA), 2 mM L-glutamine (Sigma-Aldrich, Taufkirchen, Germany), 10 U/mL penicillin, 10 mg/mL streptomycin and 100 mM sodium-pyruvate (all from Gibco Invitrogen, Grand Island, NY, USA). The cultures were subsequently incubated at 37 °C under 5% CO_2_ for 1 and 7 days (time required for HFFs to adhere and proliferate on the nanofibrous surfaces, respectively). At these time points, cell adhesion, proliferation and viability were evaluated using tissue culture polystyrene (TCPS) as positive control, as will be explained in the following sections.

### 2.5. Live/dead Assay by Fluorescence Imaging

To qualitatively evaluate cell viability, live/dead cell staining was used in combination with fluorescence imaging. Prior to staining, the supernatant was removed, the PCL samples were rinsed twice and 1 mL of phosphate buffered saline (PBS) was added to the cultures. In a next step, the samples were incubated in 2 μL (1 mg/mL) of calcein-acetylmethoxyester (Anaspec, Liege, Belgium) supplemented with 2 μL (1 mg/mL) propidium iodide (Sigma-Aldrich, Taufkirchen, Germany) to perform the staining. Using this staining step, the live cells were stained in green by calcein-acetylmethoxyester, while the dead cells were stained in a red colour by propidium iodide. Subsequently, the cultures were incubated for 10 min at room temperature in the dark, rinsed twice with PBS and then imaged with a fluorescence microscope (Olympus; IX 81 Tokyo, Japan) making use of appropriate filters. To eliminate the autofluorescence effect of the coatings, a dark background was applied, which was obtained from stained samples without the presence of cells. Moreover, the same exposure time and light intensity as used to set the dark background were applied during imaging of the cell-seeded samples. As previously mentioned, cell evaluations were performed 1 and 7 days post-seeding. 

### 2.6. MTT Assay

A colorimetric MTT assay, using the yellow tetrazolium dye 3-(4,5-dimethyldiazol-2-yl)-2, 5-diphenyltetrazolium bromide (MTT, Merck Promega, Darmstadt, Germany), was also performed to quantify cell adhesion and proliferation by colorimetrically measuring the amount of metabolically active HFFs. The tetrazolium component is reduced in viable cells by mitochondrial dehydrogenase enzymes into blue-purple water-insoluble formazan, which can be solubilized by the addition of lysis buffer and measured making use of spectrophotometry. For this purpose, the cell culture medium was first removed and replaced by 0.5 mL (0.5 mg/mL) MTT reagent. Subsequently, the cultures were incubated for 4 h at 37 °C after which the MTT reagent was replaced by a lysis buffer (1% Triton-X100 in isopropanol/ 0.04 N HCl) to solubilize the water-insoluble formazan. The exposure to this lysis buffer was conducted for 30 min at 37 °C. Afterwards, 200 µl of the formazan solution was transferred to a 96-well plate and the absorbance of the coloured solution at 580 nm was measured using a spectrophotometer (universal microplate reader EL 800, Biotek Instruments, Winooski, VT, USA). The optical density of the coloured solution at day 1 and day 7 in this work was reported as a ratio compared to TCPS at day 1 and day 7, respectively, and triplicate measurements were performed at the same time points as the microscopic evaluations (1 and 7 days after cell seeding). The obtained optical density results were subjected to one-way ANOVA and a multiple pairwise comparison test using the Tukey-Kramer method and applying a P-value of 0.05.

### 2.7. Examination of Cell Morphology by SEM

The cell morphology (shape of adhered cells and characteristics of filopodia) of the plasma-polymerized and unmodified NFs scaffolds was also evaluated 1 and 7 days after cell seeding using SEM. For this purpose, the scaffolds were gently removed from the culture media and rinsed 3 times with PBS to remove non-adhered cells. In a next step, the cells were fixed by soaking the samples in a fixative solution (2.5% glutaraldehyde in cacodylate buffer (Sigma-Aldrich, Taufkirchen, Germany)) for 1 h at room temperature. Afterwards, the samples were removed from the fixative solution and washed in cacodylate buffer, after which the cells were dehydrated by immersing the samples in increasing concentrations of ethanol (50%, 75%, 85%, 95% and 100%) for 10 min each. After dehydration, the samples were immersed in hexamethyldisilazane (HMDS, Steinhem, Germany) twice for 10 min and subsequently air-dried. Finally, the NFs with fixed, dehydrated cells were then sputter-coated with gold and imaged by SEM. To semi-quantify the cell morphology, SEM images of 2 individual cells located on 2 different samples prepared using the same plasma operational parameters were also taken, from which the cell area (A) and cell perimeter (P) were determined. These values were in turn used to calculate the cell shape factor (f_circ_) using the following equation [43]:

(4) $$f_{circ}=\frac{4\pi A}{P^{2}}$$

This shape factor is a dimensionless quantity with a maximum value of 1 in case of a perfect circle. Lower shape factors thus indicate better cell spreading. 

## 3. Results and Discussion

### 3.1. Surface Morphology

**Figure 1:**
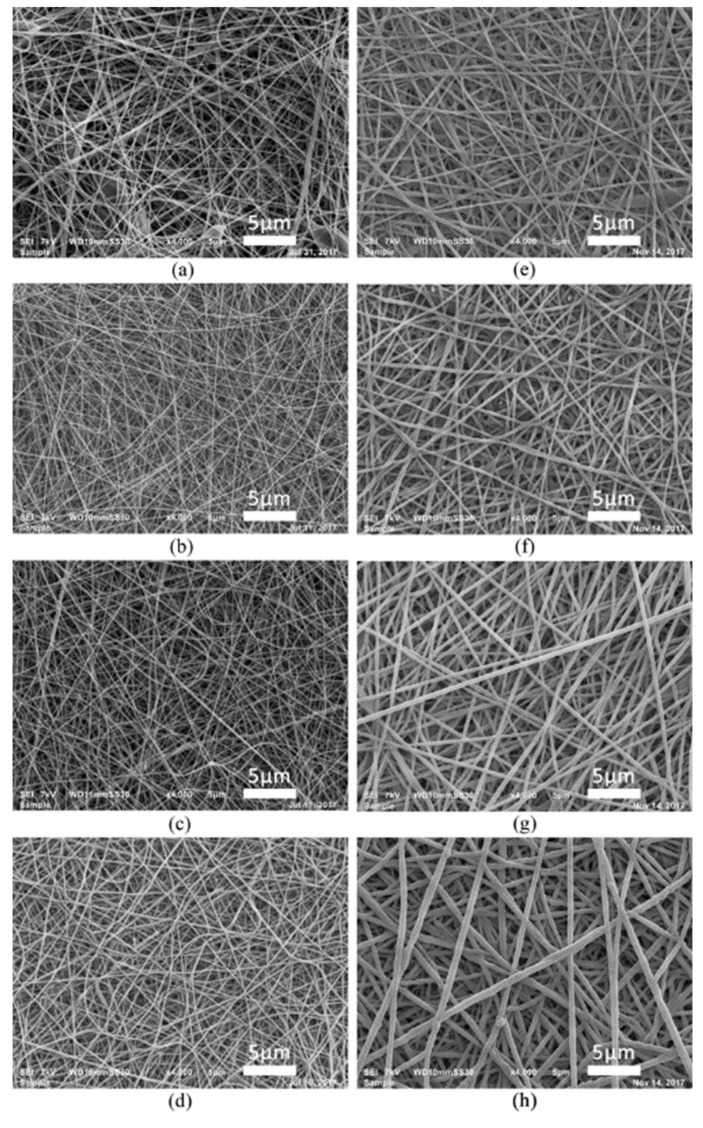
SEM images of (**a**) untreated and (**b**–**h**) PPF-NFs with various deposition durations: (**b**) 10 s, (**c**) 20 s, (**d**) 60 s, (**e**) 90 s, (**f**) 120 s, (**g**) 240 s and (**h**) 360 s.

According to literature and researchers’ experience, the surface morphology of tissue engineering scaffolds plays one of the most crucial roles in cell biocompatibility [44,45]. Thus, preserving the desirable NFs structural features is very important when performing any type of surface modification on nanofibrous scaffolds. Particularly, when depositing a functional film, adequate coating thickness is required while preserving the preferable NFs scaffolds morphology. Considering the aforementioned, this work initially focused on examining the morphological changes to the NF scaffolds as the plasma deposition duration increases. Figure 1 shows the SEM images of the untreated NFs and PPF-NFs with deposition durations varying from 10 to 360 s. It can be clearly observed that there was no drastic change in fibre morphology across all deposition durations. This implies that the plasma treatment did not inflict any damage on the NFs such as melting or fiber deformation.

**Figure 2:**
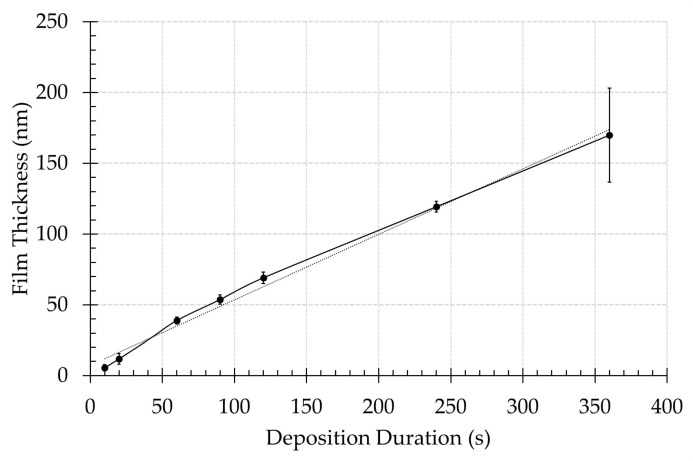
The evolution of the plasma-polymerized film thickness with plasma deposition duration.

It is also evident from Figure 1 that the diameter of the PCL NFs increases with increasing plasma deposition duration. As was already mentioned in the experimental part, ImageJ software was used to measure the diameter of the PCL NFs on the SEM images obtained for each experimental condition. These measurements confirmed that the untreated sample consists of PCL fibres possessing an average fibre diameter of 117.6 ± 22.0 nm, which are very thin nanofibers thanks to the use of the solvent mixture formic acid/acetic acid. Upon performing plasma deposition, this fibre diameter gradually increases up till 442.2 ± 86.5 nm for samples exposed to a plasma polymerization step of 360 s due to accumulation of the deposited film on the outer surfaces of the NFs. It is worth mentioning that even after the longest deposition duration under study, the obtained coated PPF-NFs scaffolds preserve their original nanomorphology. Next, Equation (3) was applied to determine the thickness of the PPFs and the results are shown in Figure 2 as a function of plasma deposition duration. It was found that the thickness of the PPFs linearly increases with deposition duration from 5.5 ± 2.1 nm to 169.9 ± 33.2 nm for a deposition time of 10 s and 360 s, respectively, resulting in a film growth rate of approximately 0.465 nm/s. This film growth rate is significantly lower than the deposition rate of CPA-based plasma polymers observed on flat substrates (4.26 nm/s) in previous work [38], which can be attributed to the very high surface area of the nanofibrous samples in comparison to a flat substrate.

### 3.2. Surface Chemical Composition

**Figure 3:**
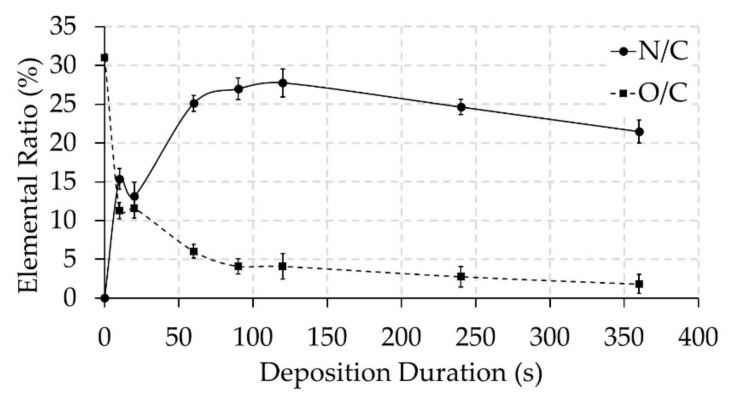
Evolution of the N/C and O/C ratio as a function of plasma deposition duration.

In the next step of this study, the PPF-NFs were subjected to XPS analysis to characterize the surface elemental composition. The results obtained from wide range XPS scans are summarized in Table 1. As expected, the untreated NFs contain only C and O elements with percentages close to the theoretical value of PCL (25% of O and 75% of C). In contrast, the surfaces of the PPF-NFs contain, besides C and O, nitrogen atoms due to the deposition of a nitrogen-rich thin film. Figure 3 shows the content of oxygen and nitrogen relative to carbon for all experimental conditions used in this work to better evaluate the elemental composition results. After 10 s of plasma polymerization, the PCL NFs surface already contains an N/C ratio of 15%, which is an obvious indicator of the PPF presence. Consequently, the O/C ratio rapidly drops from 31% to 11% for the same sample due to the coverage of the PCL NFs by a thin nitrogen-rich film. In this particular case, the film thickness is smaller (5.5 ± 2.1 nm) than the sample penetration (7–10 nm) depth of XPS, meaning that the observed oxygen is not necessary present in the film, but may also be due to the oxygen present in the underlying PCL NFs. It can be seen in Figure 3 that starting from a deposition duration of 20 s till 120 s, the N/C ratio gradually grows, while the O/C ratio further decreases. This can be explained by the growth of film thickness with deposition time, which lowers the number of oxygen photoelectrons coming from the underlying PCL NFs substrate. After reaching a peak N/C ratio of almost 28% at 120 s, the PPF-NFs experienced a gradual decrease in N/C ratio, combined with a small decrease in O/C ratio (see also Table 1). However, these decreases are relatively small in comparison to the initial increase/decrease of N/C and O/C, respectively (see Figure 3) and might be explained by extended exposure to plasma, which may cause some changes in surface chemical content due to the occurrence of a combination of etching and deposition. Table 1 and Figure 3 also reveal that the deposited PPFs mainly consist of carbon and nitrogen, with only a very small amount of incorporated oxygen, as evidenced from the results obtained at higher plasma polymerization durations. The small incorporation of oxygen can be attributed to two different processes: (1) post-plasma oxidation processes via dangling bonds and active sites during exposure to ambient air or (2) direct oxygen incorporation during the plasma deposition process due to the presence of residual air/water in the plasma chamber [46,47]. As the samples were transferred to the XPS machine within 1 hour after the plasma polymerization experiments, we anticipate that the small oxygen incorporation is mainly directly incorporated during the plasma polymerization step, as also in the case of Ar surface activation of PCL NFs small amounts of oxygen incorporation were observed using a similar plasma reactor [5]. To exactly determine the contribution of in situ oxidation and post-plasma oxidation upon exposure to ambient air, plasma-polymerized samples should be transferred directly from the plasma deposition chamber to the XPS equipment without any exposure to ambient air, which is out of scope of this particular paper.

**Figure 4:**
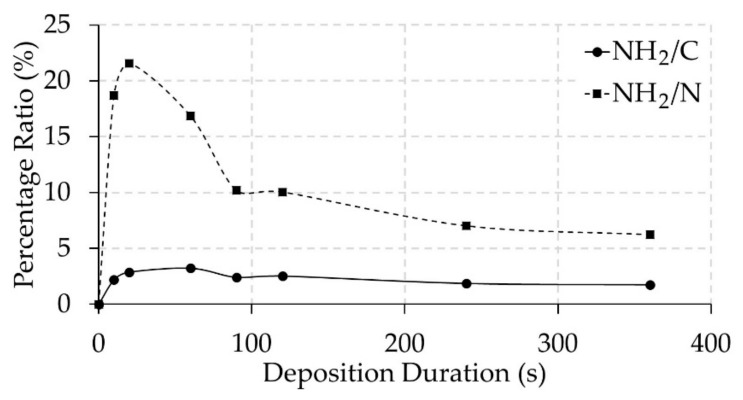
Evolution of amino grafting efficiency (NH_2_/C) and amino selectivity (NH_2_/N) as a function of plasma deposition duration.

The amino grafting efficiency and selectivity of the PPFs was also determined based on XPS analysis of TFBA-derivatized samples making use of Equations (1) and (2) [38]. The amount of incorporated F atoms which were detected on the TFBA-derivatized plasma-polymerized samples (see Table 1) clearly indicate the presence of primary amine groups on the PPFs. Similar to the N/C ratio in Figure 3, it was found that the amino grafting efficiency (NH_2_/C) experienced a strong increase from 0 to a maximum value of 3.2% at a plasma polymerization duration of 60 s, while the amino grafting selectivity (NH_2_/N) strongly increased to a peak value of 21.6% at a deposition duration of 20 s, as clearly shown in Figure 4. Moreover, two parts can be clearly distinguished on the graph: (i) the deposition duration region 20–120 s, in which differences in NH_2_/C and NH_2_/N ratio can be associated with varying coating thickness (see further) and (ii) the deposition duration region 120–360 s where a very small decrease in NH_2_/C ratio can be observed in combination with a decrease in NH_2_/N ratio till 1.8% and 6.2% respectively. These decreases may be due to a longer exposure of the deposition film to UV radiation and active species produced by the plasma, which may result in a combination of etching and deposition. The relation between the film thickness and the penetration depth of XPS and TFBA derivatization greatly influences the obtained values of amino grafting selectivity. For a PPF thickness smaller than the penetration depth of XPS, the concentration of fluorine will be higher, and it will decrease when the film thickness increases at longer deposition time. This conclusion was substantiated from a study using XPS depth profiling by Manakhov et al. [48], which demonstrated that when using TFBA derivatization, the fluorine concentration was not uniformly distributed within the deposited film. Furthermore, the deposition rate across the PCL NF samples will not be homogeneous because deeper pores will show a significantly lower deposition rate on the sample [49]. This might, in turn, create localized areas on the NF samples where the PPF thickness is smaller than the XPS penetration depth, even after using long deposition durations. The culmination of these factors can explain the initial high amino selectivity (NH_2_/N) that was observed for 10 and 20 s PPF-NFs as the ratio between the attached F and the total amount of N for these short deposition durations is much higher than for the thicker coatings (longer plasma exposure time). Furthermore, it is well known from literature that –NH_2_ groups are very effective in increasing the hydrophilicity of a surface due to their polar nature [50,51,52,53]. Also in this work, it was observed that from 10 s of deposition duration, a water droplet was rapidly absorbed into the PPF-NF mesh indicating a highly hydrophilic surface (see Supplementary Materials: Video S1 for the untreated PCL sample and Video S2 for the sample after 10 s plasma deposition)). This result thus indicates that the surface free energy of the nanofibrous samples strongly increases as a result of the plasma polymerization process.

**Figure 5:**
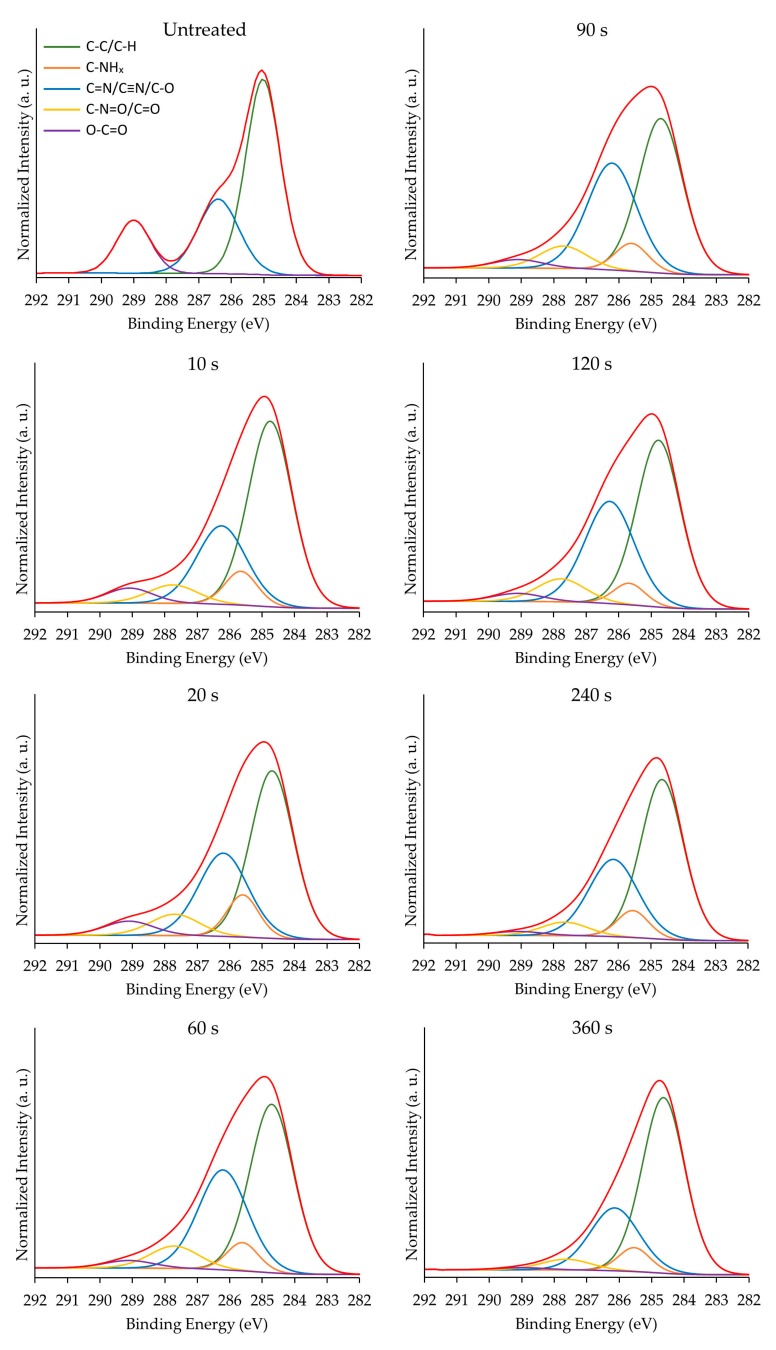
The deconvolution of XPS high resolution C1s peaks of untreated PCL NFs and PPF-NFs with a deposition duration varying between 10 and 360 s.

Additionally, to the surface elemental composition, the new surface functionalities on the PPF-NFs were also investigated as these are another crucial parameter influencing sample biocompatibility besides sample morphology. For this purpose, detailed C1s XPS spectra were deconvoluted using the following chemical bonds: C–C/C–H (C1), C–NH_x_ (C2), C=N/C≡N/C–O (C3), N–C=O/C=O (C4) and O–C=O (C5) at 285.0, 285.9, 286.5, 288.0 and 289.0 eV respectively. The selection of these chemical groups was based firstly on the PCL chemical structure and secondly on the possible CPA plasma polymerization products [38,39,54,55]. The deconvolution of the C1s peaks of untreated PCL NFs and all CPA-coated PCL NFs are shown in Figure 5.

Table 2 summarizes the quantitative chemical group results obtained from the curve fitting of the C1s detailed spectra acquired for untreated and underivatized PPF-NFs. The relative concentrations of chemical bonds defined for the pristine PCL NFs are in good agreement with earlier obtained results on the same material [5]. From these results, it is clear that with increasing deposition duration, the chemical fingerprint from the PCL NFs substrate rapidly disappears represented by the decrease of the C5 component. Simultaneously, with increasing deposition time, strong increases in C2, C3 and C4 signals can be observed attributed to the CPA plasma polymers as these become more prominent.

**Figure 6:**
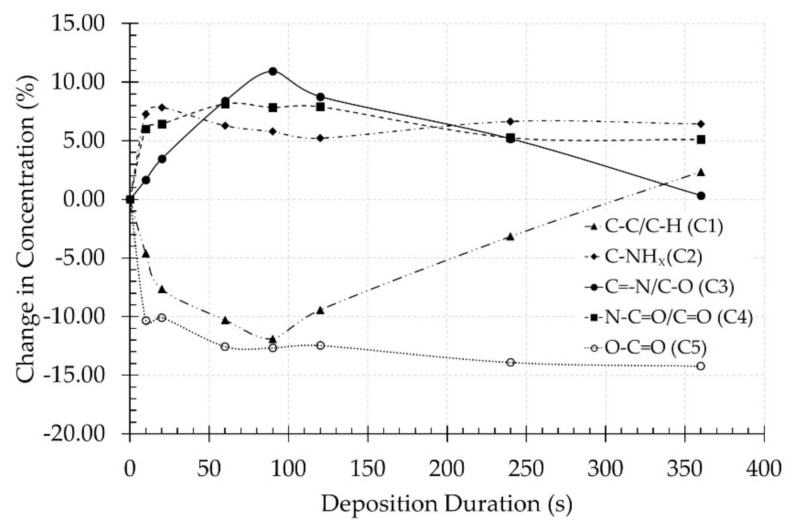
Changes in chemical bond concentrations between untreated PCL NFs and PPF-NFs as a function of plasma deposition duration.

To have a clearer observation of the aforementioned conclusions, the differences in chemical bond concentrations between untreated and plasma-polymerized scaffolds were calculated and the obtained values are presented in Figure 6 as a function of plasma deposition time. This figure clearly shows the steep increase in the concentration of nitrogen-containing functional groups (C2, C3 and C4) originating from the CPA-like thin films, which is in agreement with the evolution of the N/C ratio shown in Figure 3. Additionally, a rapid decrease in C5 concentration as a function of plasma deposition time can also be observed and this concentration reaches a plateau value between a deposition duration of 60 and 90 s, which is in agreement with the O/C ratio results presented in Figure 3. The concentration of the ester group (C5) functionality mainly originates from the PCL NFs substrates and consequently strongly decreases with increasing plasma deposition time. However, despite the fact that films thicker than the XPS penetration depth are being deposited at high plasma exposure times, a small amount of ester groups originating from the PCL substrates can still be observed on these samples due to the less-pronounced coating thickness on nanofibers located further from the sample surface in the sample bulk.

Moreover, taking into account the very low oxygen content on PPF-NFs obtained at high deposition times (see Table 1), it is also anticipated that the C3 and C4 peaks mainly originate from the presence of imines/nitriles and amides in the PPFs respectively. Furthermore, the fitting results also show a clear correlation between the C1 and C3 concentrations showing trends that mirror each other. The more imines/nitriles which are incorporated at the PPF-NFs, the less C–C/C–H functional groups are present on the coated PCL NFs. Additionally, the results also show that the evolution of the C2 concentration (C–NH_x_) is consistent with the trends observed for the amino grafting selectivity and efficiency (see Figure 4) with a maximal value at a plasma deposition time of 20 s. This is due to the fact that this C2 concentration includes primary amine groups, which have been specifically detected using TFBA derivatization in combination with XPS. Figure 6 also reveals that the concentration of the C2 and C4 bonds remains unaffected at deposition durations longer than 20 s, which could be explained by the insignificant influence of the UV radiation and plasma active species on these particular chemical functionalities (C–NH_x_ and N–C=O). In contrast, the concentration of the C1 and C3 compounds (hydrocarbons and imines/nitriles) are more strongly affected by the plasma deposition time, suggesting the higher susceptibility of these functional groups to the different plasma species. As a concluding remark, it can be stated that the deposited plasma coatings mainly consist of a mixture of amines, imines and/or nitriles and to a lesser extend amides resulting from surface oxidation.

### 3.3. Cell Adhesion and Proliferation Studies

**Figure 7:**
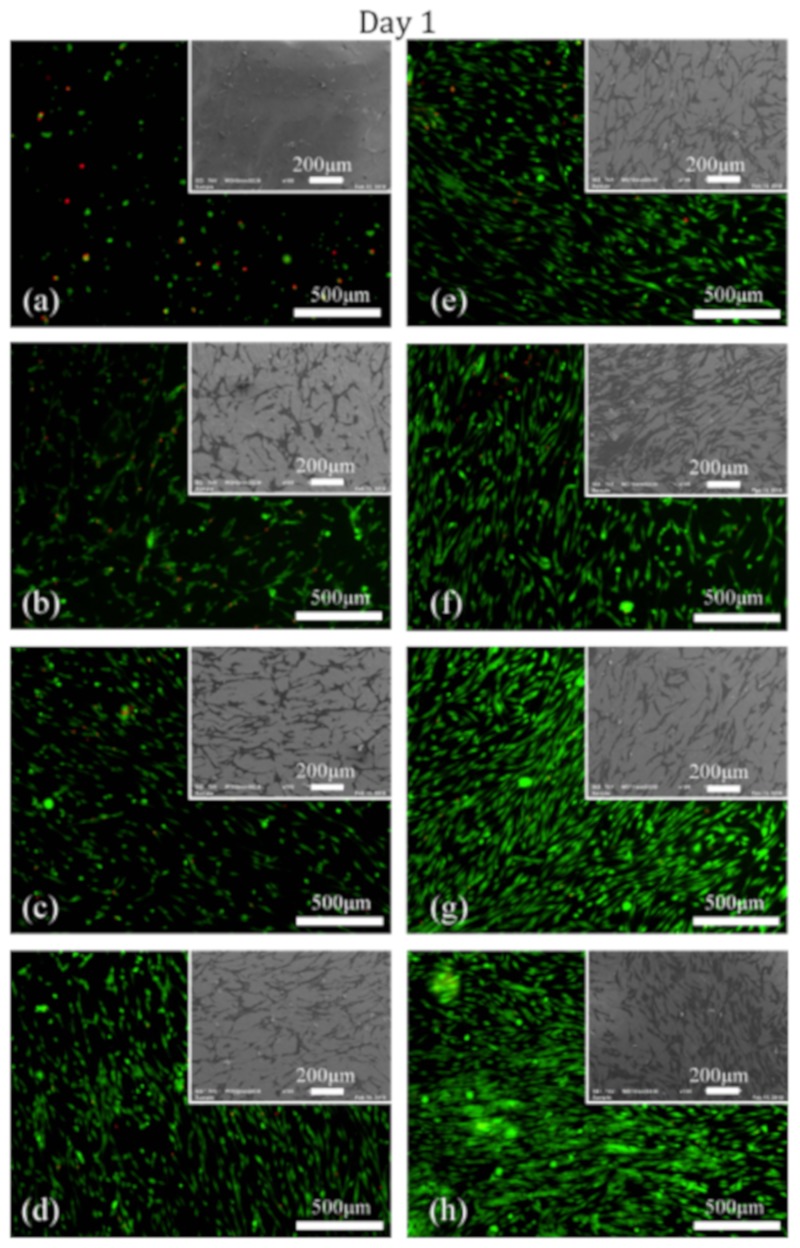
Fluorescence and SEM images of HFFs adhering on (**a**) untreated PCL NFs and (**b**–**h**) PPFs prepared using various deposition durations ((**b**) 10 s, (**c**) 20 s, (**d**) 60 s, (**e**) 90 s, (**f**) 120 s, (**g**) 240 s and (**h**) 360 s) 1 day after cell seeding.

**Figure 8:**
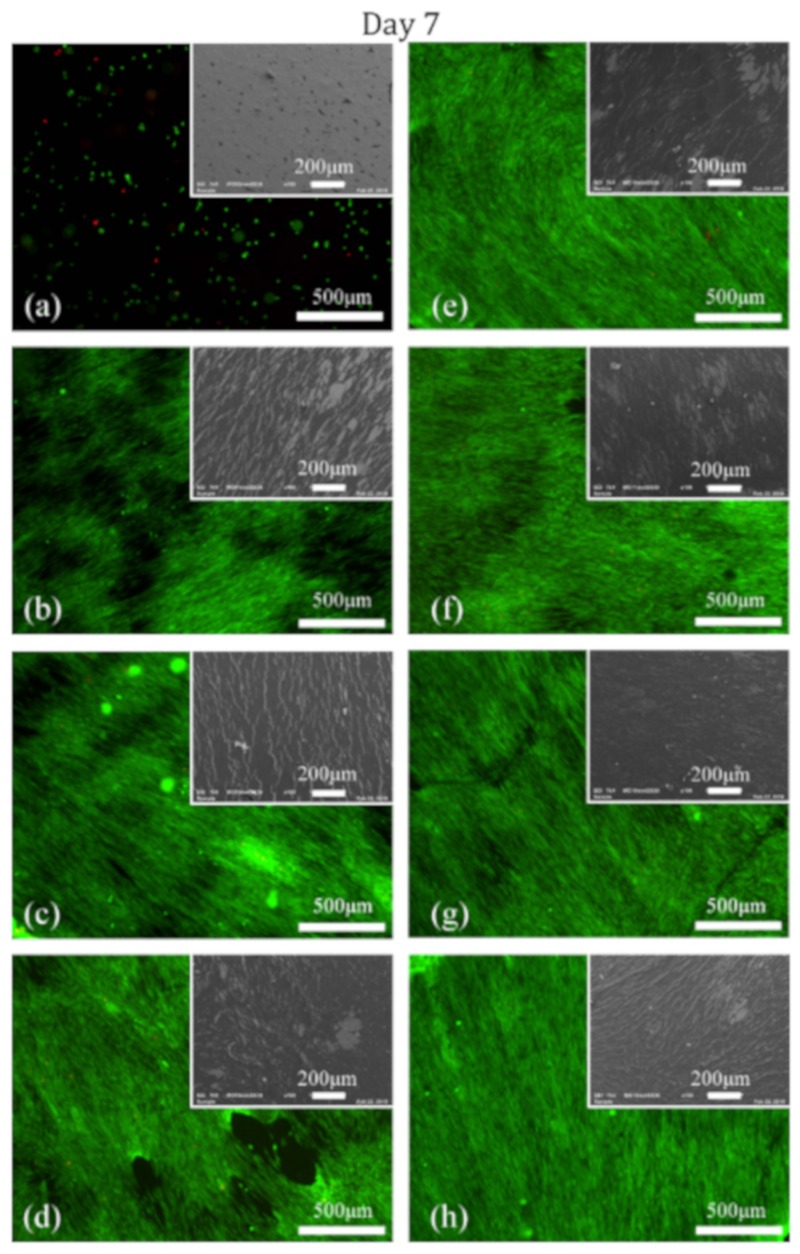
Fluorescence and SEM images of HFFs adhering on (**a**) untreated PCL NFs and (**b**–**h**) PPFs prepared using various deposition durations ((**b**) 10 s, (**c**) 20 s, (**d**) 60 s, (**e**) 90 s, (**f**) 120 s, (**g**) 240 s and (**h**) 360 s) 7 days after cell seeding.

To investigate the adhesion and proliferation of HFFs on untreated PCL NFs and PPF-NF samples prepared using different deposition durations, in vitro cell culturing experiments were performed on the following samples: untreated, 10–360 s PPF-NFs scaffolds and TCPS. These samples were evaluated 1 and 7 days post-cell seeding to examine cell adhesion and proliferation making use of live/dead staining and SEM imaging. Figure 7 and Figure 8 show the fluorescence and SEM images of untreated and differently prepared PPF-NFs 1 and 7 days post-cell seeding, respectively. As expected, poor adhesion of cells is observed on the untreated NFs on day 1, which is in stark contrast to the plasma-polymerized PCL NFs scaffolds, where excellent HFF adhesion is observed. Furthermore, it also appears that the number of adhered cells increases with plasma deposition duration as evidenced from the fluorescence images shown in Figure 7, which may be attributed to the higher coverage by the amine-rich coating of the PCL NFs at longer plasma deposition times. On the untreated sample, the HFF cells also present a round cell morphology, while the cells adopted a well-spread morphology on the plasma-polymerized samples, which is a strong indication for the enhanced cell adhesion to the substrates. Seven days after cell seeding, cellular proliferation on the untreated NFs was very poor as there was no observable increase in the number of HFFs compared to the situation 1 day after cell seeding. Additionally, the cell morphology remained round. On the other hand, the HFF cells proliferated very well on all PPF-NFs under study and an almost confluent layer of cells that can be observed at high deposition times (see Figure 8). Moreover, a huge number of viable cells with a highly-spread morphology can be seen in the confluent cell layers (see Supplementary Materials: Figure S1). This provides more evidence for excellent cell-material interaction after conducting the plasma polymerization process.

**Figure 9:**
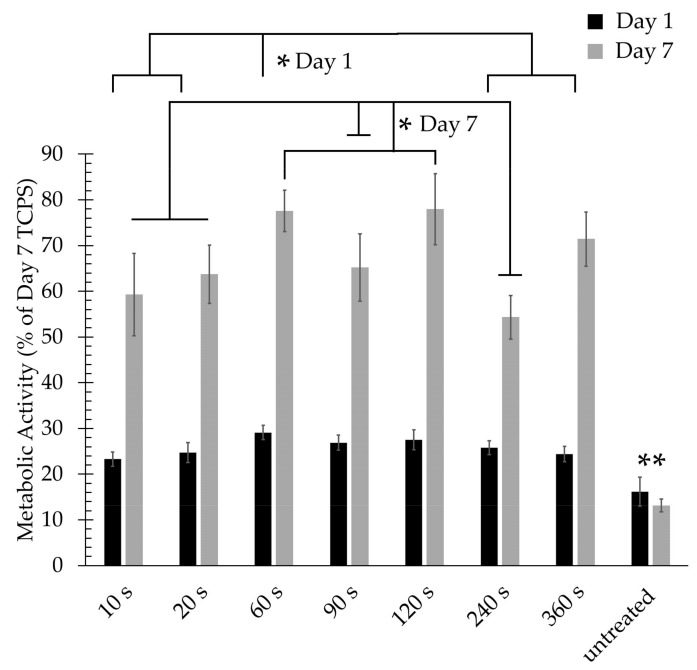
Cell viability 1 day and 7 days after cell seeding on pristine PCL NFs and PPF-NFs prepared using different plasma deposition times. The results are presented relative to the viability of TCPS on day 7 (* denotes statistical significance at * *p* < 0.05, ** *p* < 0.005).

MTT analysis was also used to quantitatively evaluate the cell viability 1 day and 7 days post-seeding; the obtained results are shown in Figure 9. The fluorescence observations made on day 1 (Figure 7) were confirmed by the MTT results showing that all PPF-NFs show significantly higher cell viability values compared to untreated NFs 1 day post-cell seeding evidencing excellent cell adhesion on the plasma-polymerized samples. When examining the influence of plasma exposure time, Figure 9 reveals that the 60 s-exposed PPF-NFs show the highest cell adhesion and this adhesion was found to be significantly higher than the cell adhesion on the 10 s, 20 s, 240 s and 360 s-exposed PPF-NFs on day 1. This conclusion could not be drawn from the fluorescence images shown in Figure 7, as these only show a qualitative overview of the cell density on a small selected area of the nanofibrous sample. In contrast, MTT analysis can provide more accurate quantitative information regarding cell adhesion and proliferation. Similarly, all PPF-NFs under study show a significant increase in optical absorbance values seven days after cell seeding compared to 1 day after seeding, which indicates a strong increased cell viability at day 7 due to the excellent proliferation behaviour of HFFs. 

In contrast, no significant increase in optical absorbance was found for untreated PCL NFs, which is in agreement with the fluorescence images presented in Figure 7 and Figure 8. Among the various tested deposition durations, the 60 and 120 s-exposed PPF-NFs show the highest absorbance values, which were significantly higher than the absorbance values on all other PPF-NFs under study, except for the 360 s-exposed PPF-NFs. Furthermore, when comparing the different cell-material analysis techniques (MTT and fluorescence imaging), the MTT results did not show a linear increasing relationship between absorbance value and deposition duration, a trend which was in contrast to that observed on the fluorescence images. This discrepancy might be explained by the fact that only a small area of the sample is visualized with the fluorescence microscope, while in case of the MTT assay, the results represent cell growth on the complete sample area. To conclude, a deposition duration of 60 s shows the best in vitro performance, which can be attributed to the fact that this coating contains a high amount of primary amines, homogenously covers the surface of the nanofibers (coating thickness of approximately 30 nm), but at the same time still preserves the delicate nanomophology of the PCL NFs. For each particular nanofibrous scaffold, the plasma deposition time should thus be optimized to obtain a full coverage of the surface of all nanofibers while maintaining the nanofibrous morphology. The optimal deposition time will thus strongly depend on the pore size and the fiber diameter of the nanofibrous scaffolds.

**Figure 10:**
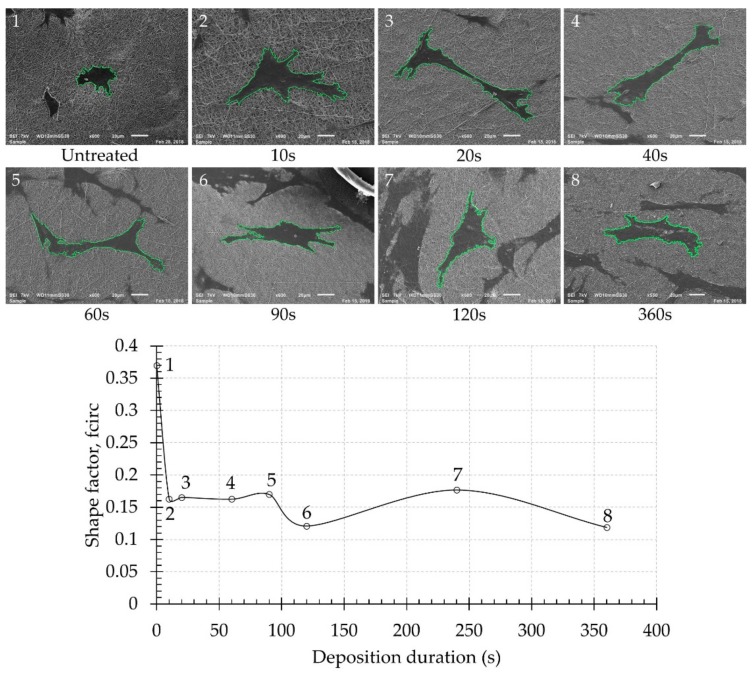
Evolution of the average shape factor of the adherent cells for the untreated and PPF-NFs with different deposition durations on day 1.

Since the degree of cell elongation or the spreading of cells can also provide information on the biocompatibility of a material, this parameter can also be used for qualitative analysis by using the circularity shape factor, which can be determined from SEM images of individual cells 1 day after cell seeding. After analysing SEM images and applying the earlier described formula (4), a shape factor was obtained for each experimental condition and its evolution as a function of deposition duration is demonstrated in Figure 10. The shape factor is directly correlated to the cell appearance and a more rounded cell morphology results in a higher shape factor value. It can be observed that the shape factor of adhered cells is significantly lower for the PPF-NFs compared to the untreated NFs, again demonstrating the strongly improved cell elongation and cell adhesion on the plasma-coated samples. Additionally, Figure 10 also reveals that the cell shape factor is not strongly affected by the deposition duration and no clear trend as a function of deposition time could be revealed, which is in agreement with the MTT results presented in Figure 9.

Interestingly, comparing the cell viability results on day 1 (Figure 10) and the C1s fitting data points of C3 (Figure 6), both parameters appear to follow a similar trend as a function of deposition duration. This observation may thus suggest that mainly the concentration of nitriles and/or imines could have a significant influence on initial HFF adhesion. This hypothesis should, however, be handled with care, as different studies have already shown that a complex surface with a variety of functional groups significantly outperforms a surface functionalised with a single type of functional groups [56,57]. Most likely, it is thus the combination of all incorporated oxygen- and nitrogen-containing functional groups which determines HFF adhesion. Furthermore, the increasing NF diameter with deposition duration can also significantly influence cell viability as the fibre diameter after 60, 90 and 120 s of deposition may be the optimal diameter range for cell adhesion of the cell line selected for this study [45].

In previous studies by Manakhov et al. [31,58], it has also been shown that CPA-based PPFs significantly improved the adhesion and proliferation of C2C12 cells on silicon substrates and PCL NFs. Although this paper has a lot in common with the work of Manakhov et al., there are still substantial differences. First of all, the plasma polymerization experiments in this work are conducted at medium pressure (50 kPa), which is significantly higher than the plasma operating pressure used by Manahkov et al. (100 Pa). Additionally, the used plasma discharge is also different: while in the present work, a 50 kHz DBD is used, Manakhov et al. used an RF discharge. As a consequence, the average discharge powers used during plasma polymerization were also quite different: in this study, a fixed discharge power of 20 W was applied, while Manakhov at al. reported discharge powers of 10 and 150 W in pulsed and continuous plasma mode respectively. These differences in pressure, power and discharge type can result in coatings possessing different chemical and physical properties as operating pressure, discharge power and discharge type can strongly affect the plasma polymerization process. Indeed, in case of a 50 nm thick coating on PCL NFs, Manakhov et al. reported NH_2_/C ratios of 2.9 and 1.5% and NH_2_/N ratios of 14 and 16% on PCL NFs, while for a similar coating thickness in this work (deposition time of 90 s), an NH_2_/C and NH_2_/N ratio of 2.5 and 10% was found. Moreover, the amount of detected O and N on the coating surface also significantly differed: in this work, it was measured to be 3.1% and 20.6%, respectively, while in the work of Manakhov et al. it was found to be 5.3%/8.9% and 14.3%/8.1%, respectively depending on the used RF mode. It is however very difficult to draw any conclusion from the comparison between both studies as completely different operational parameters (pressure, power, monomer concentration, etc.) are investigated in both papers. Nevertheless, it can be concluded that in both studies, the deposition of amino-rich coatings strongly increases cellular interactions on PCL NFs.

## 4. Conclusions

In this paper, PCL NF scaffolds were coated with a thin CPA-based plasma-polymerized film by using a sub-atmospheric pressure DBD. SEM images demonstrated the successful deposition of the CPA-based PPFs on the NFs without causing any evidential thermal damage to the nanofibrous mat morphology. The obtained results also showed that the deposited film grows practically linearly with plasma exposure time resulting in a deposition rate of 0.465 nm/s. Surface chemical analysis performed with XPS revealed an increase in N/C ratio and a decrease in O/C ratio with longer deposition durations. Moreover, it was demonstrated that the O–C=O functionalities, as a pronounced indication of the PCL NFs polymer backbone, almost fully disappeared from the NF surfaces after prolonged plasma polymerization times, while new nitrogen-containing groups (amines, amides, nitriles and/or imines) were also detected with PPF growth. TFBA derivatization in combination with XPS analysis demonstrated different trends of amino grafting efficiency and amino selectivity at shorter and longer treatment durations, which was explained by two facts: (i) PPF thickness values below the XPS penetration depth at short plasma durations and (ii) accumulating effects of exposing the sample surfaces to plasma UV radiation and active species at long plasma exposures. The amino grafting efficiency and selectivity were following the same trend as the relative percentage of amine functionalities deduced from C1s peak deconvolution on non-derivatized samples. Fluorescence images and MTT results also showed that the deposition of the CPA-based PPFs under all examined plasma exposure durations significantly improved initial attachment and proliferation of HFFs. However, no clear trend in HFF attachment and proliferation as a function of deposition time could be revealed. Nevertheless, this paper clearly proved that the deposition of an amine-rich functional coating on PCL NFs can significantly enhance cell growth on PCL NFs and may thus have important applications in the field of tissue engineering. As the primary amine functionality is known to play an important role in specific cell differentiation pathways, the current work will be extended in the near future to investigate the cell differentiation capability of the CPA-coated nanofibrous scaffolds obtained in this study.

## Figures and Tables

**Table 1:** Elemental composition of untreated and PPF-NFs for different deposition durations and percentage of incorporated F after TFBA derivatization.

Deposition Duration (s)	Before TFBA Derivatization			Incorporated Amount of F after TFBA Derivatization
	C (%)	O (%)	N (%)	F (%)
0	76.3 ± 0.4	23.7 ± 0.4	0	0
10	79.0 ± 1.0	8.9 ± 0.2	12.1 ± 0.9	4.2 ± 0.4
20	80.2 ± 0.9	9.3 ± 0.9	10.5 ± 1.5	5.3 ± 0.4
60	76.3 ± 0.9	4.6 ± 0.4	19.1 ± 1.1	5.8 ± 0.5
90	76.3 ± 1.5	3.1 ± 0.6	20.6 ± 1.0	4.5 ± 1.2
120	75.9 ± 0.8	3.1 ± 1.1	21.0 ± 0.6	4.6 ± 0.5
240	78.5 ± 1.1	2.2 ± 0.6	19.3 ± 1.0	3.6 ± 0.8
360	81.1 ± 1.1	1.5 ± 0.5	17.4 ± 0.9	3.4 ± 0.4

**Table 2:** Summary of XPS high resolution C1s peak deconvolution for untreated and PPF-NFs.

Deposition Duration (s)	C–C/C–H [C1] 285.0 eV	C–NH_x_ [C2] 285.9 eV	C=N/C≡N/C–O [C3] 286.5 eV	N–C=O/C=O [C4] 288.0 eV	O–C=O [C5] 289.0 eV
**0**	58.9 ± 0.9	-	25.8 ± 1.0	-	15.3 ± 0.4
**10**	54.3 ± 1.7	7.3 ± 1.1	27.5 ± 0.9	6.0 ± 0.4	5.0 ± 0.3
**20**	51.2 ± 1.5	9.0 ± 1.1	29.3 ± 1.5	6.4 ± 0.7	5.2 ± 0.5
**60**	48.6 ± 1.6	6.3 ± 1.2	34.3 ± 0.6	8.2 ± 0.7	2.7 ± 0.2
**90**	47.0 ± 1.6	5.8 ± 1.3	36.8 ± 2.2	7.9 ± 1.2	2.6 ± 0.3
**120**	49.4 ± 1.6	5.2 ± 0.9	34.6 ± 1.8	7.9 ± 0.3	2.8 ± 0.3
**240**	55.7 ± 1.9	6.7 ± 1.7	31.0 ± 3.6	5.3 ± 0.5	1.4 ± 0.3
**360**	61.2 ± 1.8	6.4 ± 0.6	26.2 ± 2.5	5.1 ± 0.5	1.1 ± 0.6

## Supplementary Materials

The following are available online at https://www.mdpi.com/2079-4991/9/9/1215/s1, Figure S1: Fluorescence images at higher magnification of HFFs adhering on (**a**) untreated PCL NFs and (**b**–**h**) PPFs prepared using various deposition durations ((**b**) 10 s, (**c**) 20 s, (**d**) 60 s, (**e**) 90 s, (**f**) 120 s, (**g**) 240 s and (**h**) 360 s) 7 days after cell seeding., Video S1: Water contact angle of untreated nanofiber., Video S2: Water contact angle PCL NF after 10 s plasma deposition.
