# Supplementary figures and images for: Biocompatibility of Cyclopropylamine-Based Plasma Polymers Deposited at Sub-Atmospheric Pressure on Poly (ε-caprolactone) Nanofiber Meshes
Source: Nanomaterials (Basel). 2019 Aug 28;9(9):1215. doi: 10.3390/nano9091215 (PMC6780329; doi:10.3390/nano9091215)

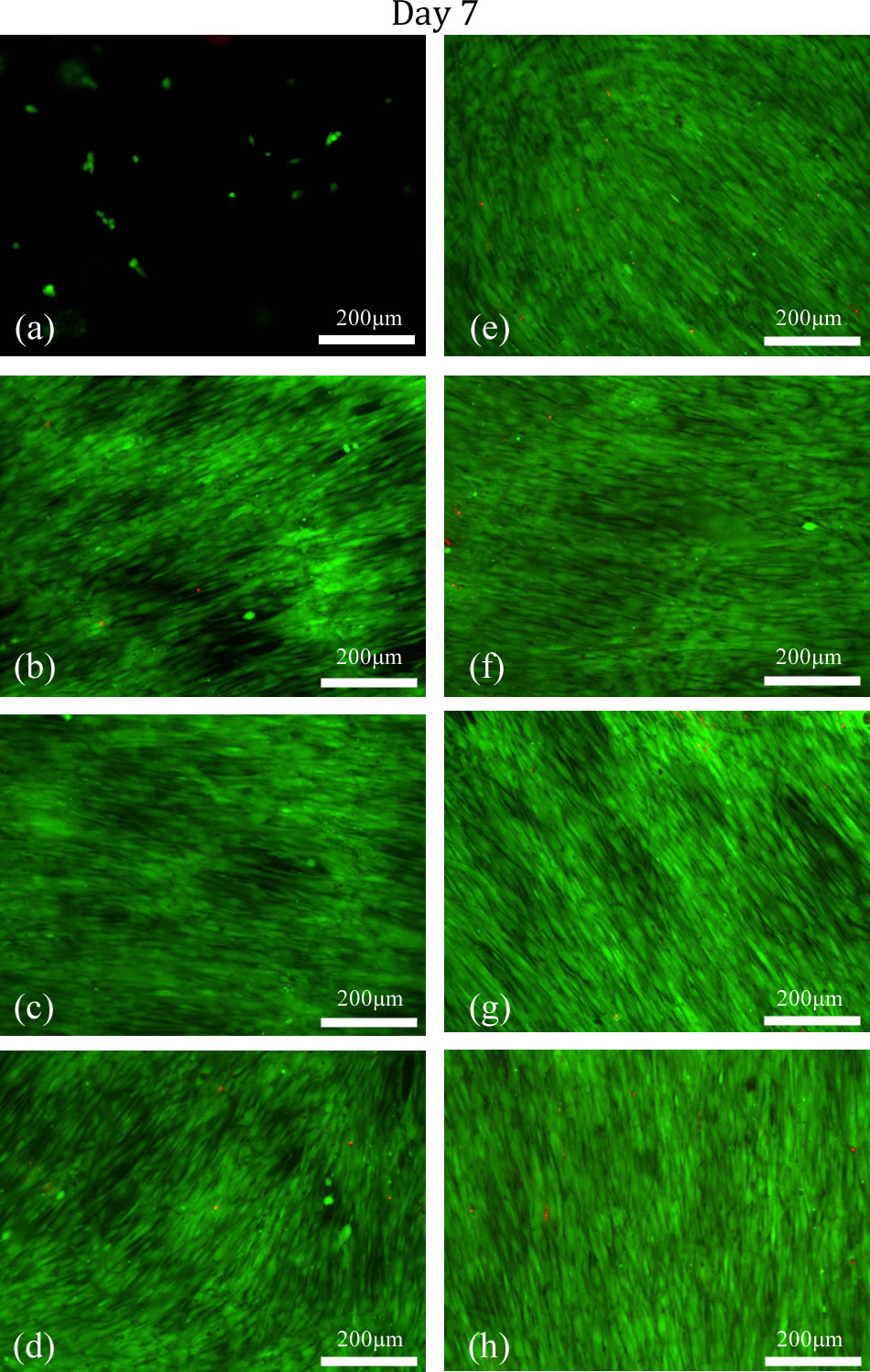

Supplement: Supplementary file 1 [file nanomaterials-09-01215-s001.zip › supplementary materials/Figure S1.jpg]
